# Physical Aggression and Mindfulness among College Students: Evidence from China and the United States

**DOI:** 10.3390/ijerph13050480

**Published:** 2016-05-10

**Authors:** Yu Gao, Lu Shi, Kelly C. Smith, Jeffery B. Kingree, Martie Thompson

**Affiliations:** 1 Division of Physical Education, Shanghai University of Finance and Economics, Shanghai 200433, China; gaoyu@mail.shufe.edu.cn; 2 Department of Public Health Sciences, Clemson Univistry, Clemson, SC 29634, USA; kingree@clemson.edu (J.B.K.); mpthomp@clemson.edu (M.T.); 3 Department of Philosophy and Religion, Clemson University, Clemson, SC 29634, USA; kcs@clemson.edu

**Keywords:** mindfulness, aggression, younger adults, adolescents, college, China

## Abstract

*Background*: The link between trait mindfulness and several dimensions of aggression (verbal, anger and hostility) has been documented, while the link between physical aggression and trait mindfulness remains less clear. *Method*: We used two datasets: one United States sample from 300 freshmen males from Clemson University, South Carolina and a Chinese sample of 1516 freshmen students from Shanghai University of Finance and Economics. Multiple regressions were conducted to examine the association between mindfulness (measured by Mindful Attention and Awareness Scale (MAAS)) and each of the four subscales of aggression. *Results*: Among the Clemson sample (*N* = 286), the mindfulness scale had a significant negative association with each of the four subscales of aggression: Hostility: β = −0.62, *p* < 0.001; Verbal: β = −0.37, *p* < 0.001; Physical: β = −0.29, *p* < 0.001; Anger: β = −0.44, *p* < 0.001. Among the Shanghai male subsample, the mindfulness scale had a significant negative association with each of the four subscales of aggression: Hostility: β = −0.57, *p* < 0.001; Verbal: β = −0.37, *p* < 0.001; Physical: β = −0.35, *p* < 0.001; Anger: β = −0.58, *p* < 0.001. Among the Shanghai female subsample (*N* = 512), the mindfulness scale had a significant negative association with each of the four subscales of aggression: Hostility: β = −0.62, *p* < 0.001; Verbal: β = −0.41, *p* < 0.001; Physical: β = −0.52, *p* < 0.001; and Anger: β = −0.64, *p* < 0.001. *Discussion*: Our study documents the negative association between mindfulness and physical aggression in two non-clinical samples. Future studies could explore whether mindfulness training lowers physical aggression among younger adults.

## 1. Introduction

Mindfulness can be considered as an inherent capacity of the human organism [1,2] that can be strengthened by both meditation and non-meditative exercise. Strengthening this capacity could decrease aggressive behavior by providing more cognitive flexibility and self-awareness, as the “experiential acceptance” [3] attitude associated with trait mindfulness might make it easier for one to receive unwelcome information in a less defensive manner [4,5,6]. Trait mindfulness was found to moderate the relation between drinking adult men’s histories of heavy (not frequent) alcohol consumption and sexual coercion/aggression, suggesting that trait mindfulness may influence the alcohol-induced disruption of executive functioning [7]. A critical review of mindfulness-based interventions to reduce violence concluded that four group studies they had reviewed found limited support for mindfulness intervention’s effectiveness while the seven single-subject studies they had reviewed displayed consistent reductions in aggressive behaviors [8]. Both this 2012 critical review of mindfulness-based intervention in reducing aggression and a 2013 appraisal of mindfulness-based interventions [9] in general have noted that limited research designs had compromised the validity of mindfulness-based interventions. As a promising attempt to reduce aggressive behavior using mindfulness-based intervention, a randomized controlled trial of 34 individuals with mild intellectual disabilities demonstrated the effectiveness of the mindfulness-based procedure for helping reduce physical and verbal aggression [10].

Although various studies have established the link between trait mindfulness and several dimensions of aggression (verbal, anger and hostility) [11], the link between physical aggression and trait mindfulness remains less clear and some of the stronger links between physical aggression and mindfulness were mainly found among the small clinical samples [12]. Moreover, important covariates that could predict aggression such as screen time are often not included in previous associational studies. The analyses were often not conducted with stratification of gender or race, partly because many studies of violence primarily focus on the male population. Finally, relatively few studies have been conducted in developing countries, which compromises the external validity of these important associational patterns.

In this study, we adopt a comparative framework to study the association between trait mindfulness and physical aggression by analyzing similar primary data of college freshmen in China and the United States. Considering that young adulthood is an age range where many violent behaviors are observed, we expect our study to have both scientific value in informing intervention studies and policy relevance in reducing aggressive behavior.

## 2. Method

### 2.1. Study Population

We used two datasets for our analyses: a United States dataset from Clemson University, South Carolina and a Chinese dataset from Shanghai University of Finance and Economics. We obtained institutional review approval from Clemson University (IRB2013-238).

The first sample was recruited from the freshman male population (1692 students as of December 2013) at Clemson University, a mid-sized public university in the southeastern United States. This sample is a part of our interventional study aimed at using mindfulness intervention to reduce alcohol misuse on college campus. We contacted all 1692 students via email and the first 300 students who logged into our online survey were allowed to participate in the initial baseline data collection to determine those at elevated risk. The baseline data were collected in January 2014, right after the Winter break. Informed consent for the baseline survey was given electronically by the respondents. Respondents were eligible to participate only if they were a freshman male physically attending the university (thus, online students were not included) at the time of the study and between 18 and 20 years of age. Respondents were also excluded from participation if they failed to give informed consent or did not understand English.

The second sample for this study covered 1516 college freshman of both genders studying at the Shanghai University of Finance and Economics (SUFE) as of December 2014, a convenience sample that constituted 75% of the school’s two thousand freshmen. This is our baseline sample of our longitudinal study of mindfulness and college health in Shanghai. A public university located in China’s financial capital of Shanghai, SUFE is a selective college with its competitive edge in business and social sciences. Students filled out the voluntary survey after their physical education classes at the end of their first semester of college. In order to be eligible for the survey, each individual had to be a freshman at SUFE who had just finished their first semester of classes and was still enrolled at the University.

### 2.2. Key Variables

A respondent’s four dimensions of aggressiveness (hostility, verbal aggression, physical aggression, and anger) were measured by the 29-item Buss and Perry Aggression Questionnaire [13]. Cronbach’s α for the four sub-scales are: hostility = 0.88, verbal aggression = 0.69, physical aggression = 0.78, anger = 0.86, respectively. The well-validated Mindful Attention Awareness Scale (MAAS) [14,15] was used to measure the mindfulness of the respondent (Cronbach’s α = 0.88). All four subscales of aggression and the MAAS scale were transformed into scales of 0 to 100 to facilitate interpretation of the regression results.

We include the students’ screen time (weekday and weekend time spent on television and non-television screen for recreational purposes) as a variable because screen time has been identified as associated with aggressive behavior [16]. Frequency of getting drunk within the 30 days (“In the past 30 days, how often did you drink enough to get drunk?”) was used as a control variable as it relates to both mindfulness [17] and aggression [18]. We used race/ethnicity as a covariate in our analysis of the Clemson University sample and urban/rural household registration status as a covariate in the SUFE sample (the latter is a determinant of health disparity in mainland China [19,20]).

### 2.3. Statistical Analysis

Four multiple linear regressions were conducted to examine the association between mindfulness and each of the four subscales of aggression. We stratified the regression analyses by gender when we estimated the association between aggression and mindfulness among SUFE students. STATA 12.0 (Stata Corporation, College Station, TX, USA) was used in these analyses.

## 3. Results

As described in Table 1, the male college freshmen sample from Clemson University were predominantly White (88.6%), while 7.1% of the sample were African Americans and 4.7% were Latinos. Only 57% of these 300 respondents reported not getting drunk in the past 30 days. On average, these students spent 18.5 h (standard deviation: 7.0) on television per week and their average MAAS score in a range from 0 to 100 is 53.9 (standard deviation = 10.4).

The descriptive statistics of the SUFE sample were documented in an earlier paper [21]. Male students made up 38.0% of the sample and the average age was 18.3 (standard deviation = 0.82) and 81.1% reported an urban household registration. The average MAAS score in a range from 0 to 100 is 64.7 (standard error = 14.06). On average, these SUFE freshmen students spent 4.78 h of screen time (standard error = 6.43) for recreational purpose per day.

Table 2 shows the results of 12 linear regressions examining the association between trait mindfulness and aggressive behaviors, among the Clemson University sample, the SUFE male subsample and the SUFE female subsample. In the four regression models using the Clemson University sample (*N* = 286), the mindfulness scale had a statistically significant negative association with each of the four subscales of aggression: Hostility: β = −0.62, *p* < 0.001; Verbal aggression: β = −0.37, *p* < 0.001; Physical aggression: β = −0.29, *p* < 0.001; Anger: β = −0.44, *p* < 0.001. The regression coefficient value of −0.29 for the mindfulness variable in the physical aggression model means that for every percentage point increase in the mindfulness scale, there is a corresponding decrease of 0.29 percentage points in the physical aggression scale.

In the four regression models using the SUFE subsample of male freshmen, the mindfulness scale had a statistically significant negative association with each of the four subscales of aggression: Hostility: β = −0.57, *p* < 0.001; Verbal aggression: β = −0.37, *p* < 0.001; Physical aggression: β = −0.35, *p* < 0.001; Anger: β = −0.58, *p* < 0.001. The three models of hostility, verbal aggression and physical aggression had an analysis sample size of 834 and the anger model had an analysis sample size of 833. The regression coefficient value of −0.35 for the mindfulness variable in the physical aggression model means that for every percentage point increase in the mindfulness scale, there is a corresponding decrease of 0.35 percentage points in the physical aggression scale.

In the four regression models using the SUFE subsample of female freshmen (*N* = 512), the mindfulness scale had a statistically significant negative association with each of the four subscales of aggression: Hostility: β = −0.62, *p* < 0.001; Verbal aggression: β = −0.41, *p* < 0.001; Physical aggression: β = −0.52, *p* < 0.001; and Anger: β = −0.64, *p* < 0.001. The regression coefficient value of −0.52 for the mindfulness variable in the physical aggression model means that for every percentage point increase in the mindfulness scale, there is a corresponding decrease of 0.52 percentage points in the physical aggression scale.

## 4. Discussion

Although the link between the lower level of mindfulness and different aspects of aggression has been documented in previous studies, our study is among the first to document the negative association between mindfulness and physical aggression in two larger samples that were not drawn from clinically diagnosed populations. These findings enrich the current literature by providing evidence from both the United States, a developed country, and mainland China, a developing country. It is notable that the associational pattern between mindfulness and four different sub-scales of aggression is consistent across the two genders and across the two very different countries, which suggests that the link between mindfulness and various aspects of aggression is a robust one at least among college students.

The fact that both samples were convenience samples limits the external validity of our study. SUFE’s high selectivity in its admission process and Clemson University sample’s regional bias as a public university further limit the generalizability of what we found from this study. Ideally, such cross-sectional analyses need to be conducted using samples that represent the college student population in a defined geographic region (country, state, city, *etc.*). We expect to work with existing population survey schemes such as the National Health Interview Survey in the United States to add mindfulness and aggression measures to the questionnaires in our future research efforts, potentially increasing the external validity of the findings in this paper.

The other limit of our study lies in the fact that the Mindful Awareness Attention Scale used here is not the only mindfulness scale currently in use and recent literature has documented some controversy of this scale [22,23,24]. According to Grossman, MAAS defines mindfulness by “how poorly I think I pay attention during everyday awareness” [22]. If so, it is hard to explain the negative association between mindfulness as measured by MAAS and physical aggression. A mindfulness measure with a multifactorial scale such as the Five Facet Mindfulness Questionnaire [25] (FFMQ, which measures five different aspects of mindfulness) might provide better insights as to precisely what aspect of mindfulness predicts aggression. For instance, if the acceptance and non-reactivity aspects of mindfulness are more important than other aspects in reducing physical aggression, then the FFMQ scale needs to be used for future studies of mindfulness and aggressive behavior.

One variable we plan to measure in future waves of the survey questionnaires in SUFE is the self-reported meditation experience among the students. Meditation practice among some of the students might have increased their trait mindfulness while decreasing aggression. The fact that we did not measure this variable and thus could not include it in the regression model might have biased the estimation toward a significant and negative association between trait mindfulness and aggression.

Finally, this study used different dimensions of self-reported aggression as the dependent variables, and we are aware that, in this study, we had no check for the fidelity in these self-reported aggression measures. The aggression scales developed by Buss and Perry [13] is a validated scale but might not be free of social desirability bias. For future studies, survey designs that collect more objective measures for aggressive behavior (e.g., teacher-reported aggressive tendency of students) might help address the social desirability bias.

Given the limits of cross-sectional data, we cannot make causal inference from these associational patterns. From the published literature, the causal mechanism behind the negative association between aggression and mindfulness could be that more mindful people have more response inhibition [26] (as evidenced by functional MRI) or more control over behavioral dysregulation [27]. Other possible explanatory mechanisms include decentering [28], reduction of rumination [29], metacognitive insight [30,31], cognitive flexibility [4,7], all of which have been shown as associated with mindfulness in documented evidence.

## 5. Conclusions

According to recent findings from mindfulness-based interventions [32,33,34], these sets of attention-based, regulatory, and self-inquiry training regimes have proved effective in treating addictive disorders in substance abuse. If the causal link between trait mindfulness and physical aggression can be proven in future studies among the nonclinical populations of adolescents and younger adults, mindfulness training could have substantial potential to reduce physical aggression and its related health burden at the population level. Teaching mindfulness-related skills such as mindfulness-based stress reduction [35] in the form of “leisure skills” to the nonclinical population on campus might be helpful in reducing aggressive behavior, and thus foster a safer environment for college students.

## Figures and Tables

**Table 1:** Descriptive statistics of 286 male freshmen from Clemson University.

Variables	Mean Value (Standard Deviation in Parentheses)	Frequency
Trait mindfulness as measured by MAAS (range: 0–100)	53.9 (10.4)	
TV hours per week	18.5 (7.0)	
Race/ethnicity		
Non-Latino White		83.6%
African American		7.1%
Latino		4.7%
Other		4.7%
Frequency of getting drunk last 30 days	1.8 (1.1)	

MAAS: Mindful Awareness Attention Scale.

**Table 2:** Mindfulness and four sub-scales of aggressiveness: linear regressions from two college freshmen samples.

Sample	Hostility	Verbal Aggressiveness	Physical Aggressiveness	Anger
	Regression Coefficients (Standard Error in Parentheses)			
Clemson male (*N* = 286)	−0.62 *** (0.06)	−0.37 *** (0.06)	−0.29 * (0.05)	−0.44 *** 0.06)
SUFE female (*N* = 834)	−0.57 *** (0.03)	−0.37 *** (0.04)	−0.35 *** (0.04)	−0.58 *** (0.04)
SUFE male (*N* = 512)	−0.62 *** (0.04)	−0.41 *** (0.05)	−0.52 *** (0.06)	−0.64 *** (0.06)

* *p* < 0.05, *** *p* < 0.001. SUFE: Shanghai University of Finance and Economics. Note: The Clemson University model controls for age, gender, race/ethnicity, weekly hours spent on television viewing, and frequency of getting drunk in the past 30 days. The Shanghai University of Finance and Economics models controls for age, gender, urban household registration, hours of recreational screen time per day and frequency of getting drunk in the past 30 days.
